# Prevalence of Seizures in Patients With Tuberculous Meningitis (TBM) and Their Clinical Outcomes

**DOI:** 10.7759/cureus.77210

**Published:** 2025-01-10

**Authors:** Shyamal N Dharmana, Neeraj Singla, Kusum Sharma, Manish Modi, Manoj Goyal

**Affiliations:** 1 Internal Medicine, Postgraduate Institute of Medical Education and Research, Chandigarh, IND; 2 Microbiology, Postgraduate Institute of Medical Education and Research, Chandigarh, IND; 3 Neurology, Postgraduate Institute of Medical Education and Research, Chandigarh, IND

**Keywords:** alerted mental status (ams), cerebrospinal fluid (csf), electroencephalography (eeg), gcs (glasgow coma score ), generalized tonic-clonic seizures (gtcs), modified rankin scale (mrs), neuro mri, seizures, tuberculous meningitis (tbm)

## Abstract

Background

The diagnosis of tuberculous meningitis (TBM) is challenging, as it requires a high level of clinical suspicion and robust efforts to manage complications.

Aim

The study focussed on the early detection of seizures and their functional and final outcome with etiological workup radiologically and on lab investigations.

Design and methods

This was a prospective observational study, in which 100 suspected TBM patients were enrolled based on Lancet consensus criteria at the Postgraduate Institute of Medical Education and Research (PGIMER), Chandigarh, from August 2022 to December 2023. Patient’s clinical characteristics, TBM severity assessment, CSF analysis, cerebral imaging, and EEG were done. The modified Rankin Score (mRS) assessed clinical outcomes.

Results

The patients' mean age was 36.12 years, with the majority comprising the female gender (55%). Among the clinical presentations, the most common were fever (88; 88%), headache (60; 60%), altered mental status (AMS) (84; 84%), and seizures on presentation (29; 29%). Of the patients with seizures, 72.4% presented with altered mental status with a p-value of 0.043. Based on the severity of the presentation, 44 (44%) presented in stage II, 35 (35%) presented in clinical stage III, and the rest (21; 21%) in stage I. The GeneXpert Ultra test was positive in 50 (50%) patients for a definitive diagnosis of tuberculous meningitis. On MRI brain, infarcts (42; 42%); hydrocephalus (32; 32%), tuberculomas (31; 31%), and exudates (13; 13%) were seen in patients. Cerebral vasculitic infarcts were found to be an independent predictor of seizures (p 0.01). Out of 29 patients with seizures, 14 patients had an mRS score of 6 and 7 patients had an mRS score of <= 2 at the end of 3 months.

Conclusion

Vasculitic infarcts in TBM were strongly associated with seizure occurrence.

## Introduction

Tuberculosis is one of the leading communicable diseases in the world. Tuberculous meningitis (TBM) is one of the manifestations of tuberculosis that makes up 1-2% of all TB cases in low HIV prevalence areas; nevertheless, in areas where HIV is common, a greater proportion of TB cases are seen [1]. Tuberculosis persists as a major global health issue, and every year, around 5,00,000 patients die of tuberculosis [2]. Clinical features of TBM include fever, headache, vomiting, impaired consciousness, focal neurological deficits, and seizures.

Seizures are one of the common clinical features seen in TBM and may develop at any time during the disease course. They can present as acute symptomatic or unprovoked seizures. Acute symptomatic seizures usually manifest within the first two weeks, subside once the acute infection is over, and have less tendency to recur. Unprovoked seizures occur after the acute phase of TBM and tend to recur [3]. Seizures are one of the serious manifestations of TBM that cause a heavy burden in the form of seizure-related disability, mortality, comorbidities, stigma, and costs. Seizures were classified based on the clinical manifestations following the International League Against Epilepsy (ILAE) 2017 classification proposal [4]. Based on frequency, seizures were classified as single seizures, recurrent seizures, or status epilepticus. A retrospective analysis of patients with TBM in China who were enrolled between 2012 and 2018 had 20.6% seizure incidence and patients with multiple seizure episodes were associated with higher mortality compared to a single episode of seizure [5].

The etiology of seizures in TBM has been attributed to multiple factors, such as meningeal irritation, cerebral edema, tuberculoma, infarction, hydrocephalus, and hyponatremia, independently or in combination. A study conducted on seizures in TBM had an incidence of 34.2%. Seventy point four percent (70.4%) of these seizures had occurred after one month. Late seizure presentations are commonly observed with tuberculoma, infarction, and hyponatremia [6].

This study primarily emphasizes the early detection of seizures in patients with TBM based on clinical history, the electroencephalogram (EEG), and treatment that can significantly alter the neurological outcome of the patient.

## Materials and methods

The study was based on a prospective collection of data of 100 patients older than 12 years, admitted at a tertiary institute during a period of study from August 2022 to December 2023 admitted in emergency department and medical wards. Suspected TBM patients who fulfilled Lancet Consensus Criteria were enrolled in the study and their cerebrospinal fluid (CSF) was collected. The collected CSF sample was sent to the Department of Microbiology for microbiological-based diagnosis for TBM by the Gene Xpert Ultra method.

Inclusion criteria

The inclusion criteria were: 1. Patients above 12 years of age admitted to medical wards; 2. Patients with TBM as per Lancet Consensus Criteria

Exclusion criteria

The exclusion criteria were: 1. Patients with underlying seizure disorder/epilepsy due to causes other than TBM; 2. Patients with bacterial meningitis or other causes of meningitis; 3. Patients who have not given consent for participation in the study. 

On the initial evaluation of patients, a detailed history was taken, including symptoms of raised intracranial pressure, seizures, and vomiting were recorded in case record form. Comorbidities such as diabetes, hypertension, coronary artery disease, and a history of tuberculosis were recorded. Conscious levels were analyzed by the Glasgow Coma Scale (GCS) score and the TBM Severity Score. Clinical examination was focused on focal or generalized seizures or any abnormal body movements. TBM is classified into three grades of severity according to the British Medical Research Council TBM grade: Grade 1 TBM is defined as a Glasgow coma score (GCS) of 15 with no focal neurology; Grade 2 TBM as a GCS of 15 with a focal neurological deficit, or a GCS of 11-14; Grade 3 TBM is defined as a GCS of ≤10.

Suspected TBM patients underwent routine blood investigations, such as complete hemogram, serum sodium, potassium levels, renal function tests, and liver function tests, and underwent brain imaging such as non-contrast computed tomography (CT) of the head at admission followed by magnetic resonance imaging (MRI) of the brain to look for hydrocephalus, vasculitic infarcts, basal exudates, and gyral enhancement.

Patients who had a history of seizure episodes and altered mental status after stabilization underwent EEG for the detection of latent seizure activity.

Patients in whom seizures were detected clinically or on EEG were followed for upto three months and patients with recurrent seizure episodes were subjected to repeat MRI of the brain for new onset infarct/ exudates. Seizure frequency, duration, and drug compliance were noted and mortality rate was calculated at the end of three months.

Statistical analysis

Data was entered in a Microsoft Excel sheet (Microsoft Corporation, Redmond, WA, US) after collection from the study participants and standardized for statistical analysis by clearance of data entry-related errors. Data were described in terms of range, mean ± standard deviation, median, and frequencies as appropriate. The Kolmogorov-Smirnov test was used to determine whether data distribution was normal. Comparison of quantitative variables between the study groups was done using the student's t-test and Mann-Whitney U test for independent samples for parametric and non-parametric data, respectively. For comparing categorical data, the chi-square test was performed. Covariates obtaining a p-value of <0.05 in the univariate analysis were included in the multivariate binary logistic regression analyses. A probability value (p-value) of less than 0.05 was considered statistically significant. All statistical calculations were done using SPSS version 21 (IBM Corp., Armonk, NY, US).

## Results

The study analyzed demographics, clinical features, investigations, imaging findings, and outcomes to understand TBM manifestations and predictors of seizures.

Demographics

The mean age of participants was 36.12 years, with a range of 13 to 84 years. Female patients accounted for 55% of the cohort.

Clinical features

Presenting Symptoms

The most common symptom was fever (88%) with a mean duration of 41.1 days, followed by headache (60%, mean duration: 19.46 days) and vomiting (29%), which has been elaborated in Table 1.

**Table 1:** Comparison of baseline clinical, laboratory, and neuroimaging parameters of patients with tuberculous meningitis with seizures and without seizures TBM: tuberculous meningitis; CSF: cerebrospinal fluid; CBNAAT: cartridge-based nucleic acid amplification test

Variable	TBM patients (n=100)	With seizures (n=29)	Without seizures (n=71)	Chi-square test	P-value
Age Mean ± SD (years)	36.12 ±16.40	35.21±17.32	36.49±16.11	-0.589	0.556
Gender Male	55 (55%)	13 (13%)	42 (42%)	1.708	0.191
Female	45(45%)	16 (16%)	29 (29%)	1.708	0.191
Fever (Duration) Days	31.99	63.52±87.93	31.99±38.99	-1.341	0.180
Headache (Duration) Days	15.28	29.69±54.41	15.28±28.19	-0.877	0.380
Altered Sensorium	84 (84%)	21 (72.4%)	63 (88.7%)	4.08	0.043
Meningeal Signs	98 (98%)	28 (96.6%)	70 (98.6%)	0.002	0.961
Focal Deficits	23 (23%)	10 (34.5%)	13 (18.3%)	3.041	0.115
Cranial Nerve Palsy	5 (5%)	0	5 (7%)	2.15	0.115
Stage I TBM	21 (21%)	5 (17.2%)	16 (22.5%)	1.012	0.603
Stage II TBM	44 (44%)	15 (51.7%)	29 (40.8%)	1.012	0.603
Stage III TBM	35 (35%)	9 (31%)	26 (36.6%)	1.012	0.603
Imaging Variables					
Basal Exudates	13 (13%)	7 (24.1%)	6 (8.5%)	4.48	0.034
Hydrocephalus	32 (32%)	9 (31%)	23 (32.4%)	0.017	0.895
Tuberculomas	31 (31%)	12 (41.4%)	19 (26.8%)	2.057	0.162
Infarcts	42 (42%)	17 (58.6%)	25 (35.2%)	4.632	0.031
CSF Variables					
CSF CBNAAT Positive	50 (50%)	13 (44.8%)	37 (52.1%)	7.266	0.122
Rifampicin Resistance	1 (1%)	1 (3.4%)	0	3.878	0.275
CSF Protein	91 (91%)	26 (89.7%)	65 (91.5%)	0.090	0.764
CSF Sugar (<40mg/dl)	64 (64%)	18 (62.1%)	46 (64.8%)	0.066	0.797
CSF Cells (Lymphocyte Predominance)	92 (92%)	26 (89.7%)	66 (93%)	0.305	0.581
Serum Sodium meq/l	131.65 ±7.64	129.97±7.85	132.34±7.49	6.543	0.259
Hyponatremia (<135 meq/L)	68 (68%)	22 (75.4%)	46 (64.8%)	4.15	0.246

**Figure 1:**
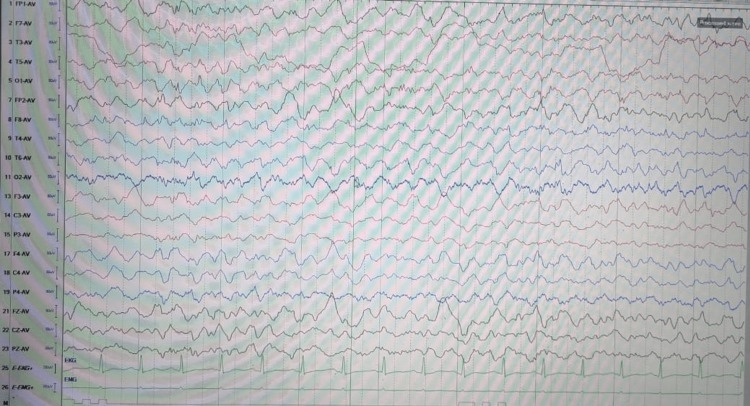
Diffuse delta slowing activity (2-3 HZ) in a patient with tubercular meningitis

Early-onset seizures (≤7 days from admission) occurred in 13% of cases and late-onset seizures in 16%, and a comparison between the two types is described in Table 2. Twenty-nine percent (29%) of patients experienced seizures, of which 25% had generalized tonic-clonic seizures (GTCS) and 4% had focal seizures, the characteristics of which are explained in Table 3. Seizure activity is usually in the form of generalized slowing or a delta wave slowing pattern as depicted in Figure 1.

**Table 2:** Comparison of TBM patients with early and late seizures TBM: tuberculous meningitis; CSF: cerebrospinal fluid; CBNAAT: cartridge-based nucleic acid amplification test; EEG: electroencephalogram

	Total TBM patients (n=100)	Early seizures (n = 13)	Late seizures (n = 16)	Chi-square Test	p-value
Age (Years)	36.12 ±16.40	41.54±18.95	30.06±14.49	-1.645	0.100
Gender - Male	55 (55%)	7 (53.8%)	6 (37.5%)	0.775	0.379
Female	45 (45%)	6(46.2%)	10(62.5%)	0.775	0.379
Imaging Variables					
Basal Exudates	13 (13%)	2 (15.4%)	5 (31.2%)	0.986	0.321
Hydrocephalus	32 (32%)	3 (23.1%)	6 (37.5%)	0.697	0.404
Tuberculomas	31 (31%)	3 (23.1%)	9 (56.2%)	3.254	0.13
Infarcts	42 (42%)	6 (46.2%)	11 (68.8%)	1.51	0.274
CSF Variables					
CSF CBNAAT Positive	50 (50%)	6 (46.2%)	7 (43.7%)	1.961	0.581
Rifampicin Resistance	1 (1%)	0	1 (6.2%)	2.466	0.481
CSF Protein (>100 mg/dl)	91 (91%)	12 (92.3%)	14 (87.5%)	0.179	0.672
CSF Sugar (<40 mg/dl)	64 (64%)	7 (53.8%)	11 (68.8%)	0.677	0.411
CSF Cells (Lymphocyte Predominance)	92 (92%)	11 (84.6%)	15 (93.8%)	0.645	0.422
EEG Variables					
Abnormal EEG	55 (55%)	9 (69.3%)	8 (50.1%)	4.355	0.226
Spike Wave Discharge	1 (1%)	1 (7.7%)	0	4.355	0.226
Generalized Slowing	28 (28%)	2 (15.4%)	5 (31.2%)	4.355	0.226
Delta Slowing	26 (26%)	6 (46.2%)	3 (18.8%)	4.355	0.226

**Table 3:** Details of seizures and electroencephalographic evaluation in TBM patients (n = 100) TBM: tuberculous meningitis; GTCS: generalized tonic-clonic seizures; EEG: electroencephalogram

Variables	Values
Seizures	29 (29%)
-Focal	04 (8.3%)
-Focal to bilateral	0
-GTCS	25 (25%)
Early onset seizures	13 (44.8%)
Late-onset seizures	16 (55.17%)
Seizures before admission	29 (100%)
Seizures during hospital stay	0
Seizures after discharge	02 (6.89%)
Acute repetitive seizures	0
Status epilepticus	0
Recurrence of seizures	02 (6.89%)
EEG variables abnormal	Values 55 (59.7%)
-Spike and wave discharges	01 (1.81%)
-Generalized slowing	28 (50.90%)
-Delta slowing	26 (47.27%)

Neurological Findings

Altered mental status (AMS) was seen in 84% of patients, 23% had neurological deficits, and Kernig’s sign was positive in 41%. Neck rigidity was observed in 98%, but only 2% had Brudzinski’s sign. Cranial nerve involvement affected 15% (most commonly the third cranial nerve).

Investigations

CSF Analysis

The mean total cell count was 304 cells/mm³, with a protein concentration of 304.8 mg/dL and glucose of 37.23 mg/dL. Lymphocytic predominance (92%) and glucose <40 mg/dL (64%) were common. No positive results for Gram stain, Ziehl-Neelsen stain, or India ink were appreciated in samples.

GeneXpert Ultra

*Mycobacterium tuberculosis bacteria *(MTB) were detected in 50% of patients. Among these, 26% were rifampicin-sensitive, 1% resistant, and 22% had indeterminate results.

Blood Investigations

The mean hemoglobin was 11.51 g/dL, sodium 131.6 mmol/L, and potassium 3.9 mmol/L. Severe hyponatremia was more common in seizure patients (24.1%).

Imaging Findings

**Figure 2:**
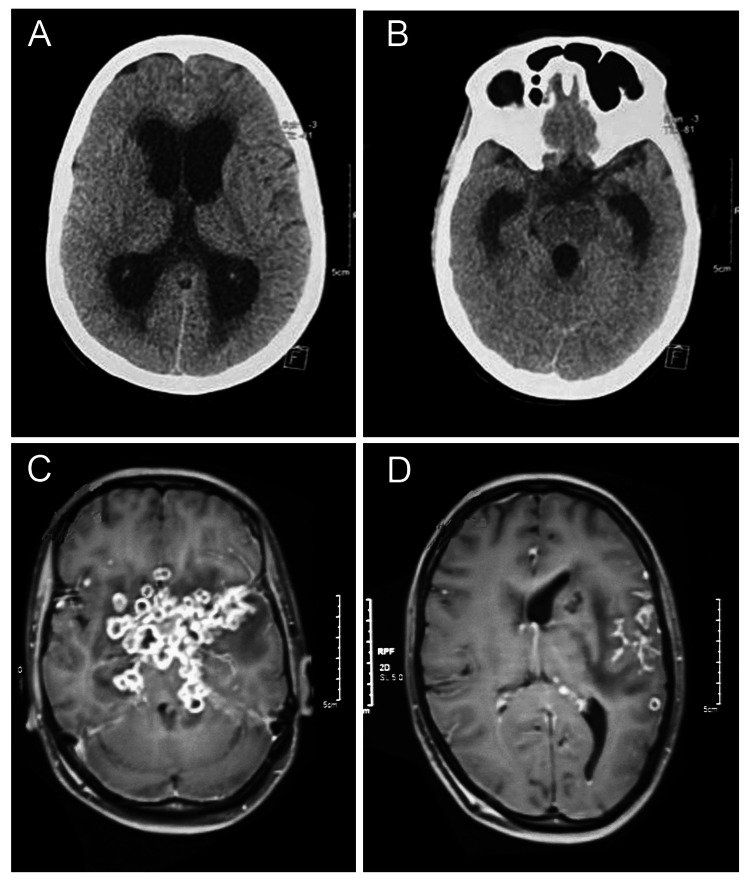
A. Non-contrast CT head axial section: dilatation of lateral and third ventricles suggestive of hydrocephalus; B. Non-contrast CT head axial section: dilatation of temporal horns of lateral ventricles and cerebral aqueduct; C. MRI brain gadolinium-enhanced T1 axial section: basal exudates with opto-chiasmatic arachnoiditis engulfing the base of the brain; D. MRI brain gadolinium-enhanced T1 axial section: leptomeningeal enhancement with thick exudates along the left Sylvian fissure

Chest X-rays showed features of pulmonary TB in 14% of cases, with a miliary pattern in 3%. CNS imaging revealed hydrocephalus (32%), infarcts (58.6% in seizure patients), exudates (24.1%), and tuberculomas (41.4%). Infarcts and exudates were significantly associated with seizures as highlighted in Figure 2.

Outcomes

Mortality

At 3 months, 43% of patients had died, 52% were alive, and 5% were lost to follow-up as described in Table 4. Mortality was higher among patients with seizures (48.3%) than those without (40.8%).

**Table 4:** Comparison between survivors and non-survivors in TBM patients with seizures TBM: tuberculous meningitis

Parameters	Characteristics	Final outcome				Total	Chi-square value	p-value
		Survivor		Non-Survivor				
Seizures (N=29)	Early onset	7	46.7%	6	42.9%	13	0.042	0.837
	Late-onset	8	53.3%	8	57.1%	16		
EEG	Normal	9	60.0%	3	21.4%	12	6.894	0.075
	Generalized theta slowing	3	20.0%	4	28.6%	7		
	Epileptic activity seen	1	6.7%	0	0.0%	1		
	Diffuse delta range slowing	2	13.3%	7	50.0%	9		
Follow-Up Modified Rankin Scale (mRS) score	0	1	6.7%	0	0.0%	1	29	0.001
	1	3	20.0%	0	0.0%	3		
	2	3	20.0%	0	0.0%	3		
	3	6	40.0%	0	0.0%	6		
	5	2	13.3%	0	0.0%	2		
	6	0	0.0%	14	100.0%	14		
Ventriculo-Peritoneal Shunting	not done	14	93.3%	12	85.7%	26	0.453	0.501
	done	1	6.7%	2	14.3%	3		
Total		15	100.0%	14	100.0%	29		

Seizure Recurrence

Recurrence occurred in 6.9% of seizure patients during follow-up, all with late-onset seizures.

Functional Outcomes

Poor outcomes (mRS ≥3) were seen in 76.9% of early-onset seizure patients and 75% of late-onset seizure patients.

Predictors of Seizures

The multivariate analysis, highlighted in Table 5, identified infarcts as an independent risk factor for seizures (p=0.01). Late-onset seizures were associated with longer fever duration, higher protein levels, and more frequent infarcts and tuberculomas on imaging.

**Table 5:** Multi-variate regression analysis with odd's ratio for mortality in TBM patients TBM: tuberculous meningitis

Parameters	p-value	Odds ratio	95% Confidence interval for odds ratio	
			Lower	Upper
Altered Mental Status	0.368	2.818	0.296	26.848
Dosage of Anti-Tubercular Therapy (ATT)	0.223	3.776	0.445	32.026
Motor Power - Normal	0.018	0.104	0.024	1.006
Motor Power - Abnormal	0.362	0.304	0.024	3.937
Motor Power Not Assessed But Moving All 4 Limbs	0.009	25.309	2.243	285.561
Involuntary Movements	0.001	581.516	32.124	10526.876
Exudates	0.134	9.678	0.497	188.426
Infarcts	0.724	0.694	0.091	5.279
Lancet Score	0.840	0.944	0.538	1.655
SGOT	0.353	1.003	0.997	1.008

## Discussion

The Global Tuberculosis Report 2018 estimates the incidence of tuberculosis to be 10 million cases, which is equivalent to 133 cases per 1,00,000 people [7]. India is one of the six high-burden countries in the Southeast Asia region, which accounts for 28% of the global tuberculosis burden. According to the National TB prevalence survey done in 2021, the crude prevalence of tuberculosis infection was around 30% in individuals aged older than 15 years [8]. Tuberculous meningitis is the most disabling form with more than 50% death or residual neurological deficits in survivors.

Seizures are one of the common findings in patients with TBM associated with an increased risk of neurological disability. In this study, seizures were present in 29 patients (29%) in concordance with the Misra et al. study [6], in which seizures were present in 34.2% of patients with TBM and in contrast to the Song et al. retrospective study on TBM [5], in which seizures were present in 20.6% of patients.

In a study done on children below 12 years with TBM by AK Patwari et al., seizures were present in 74% of patients due to immaturity of the brain [9]. This was in contrast to our study in which prevalence was found to be 29%, as the mean age of our patients was 34.5 years.

In this study, 50 (50%) of the patients had a definitive diagnosis of TBM according to Lancet Consensus Criteria in contrast to studies like UK Misra et al. [6], which had 39.2% of participants with a definitive diagnosis. This is predominantly due to the usage of Gene Xpert Ultra, which has higher sensitivity than the previously used TB Gene Xpert for the detection of Mycobacterium Tuberculosis bacilli in the CSF samples. In the study by Song et al., 49.3% of the patients were in stage I, and the rest were in stages II and III [5]. This is in contrast to our study, as only 21 % presented in stage I, as our institute was a tertiary care center that received mostly complicated cases of TBM. The other reason was the COVID-19 pandemic, which jeopardized outpatient services and patients might have neglected the initial signs and symptoms of TBM.

The clinical profile in this study was similar to the AM Wani et al. study with 68 patients, in which 84% of TBM patients had fever [10], similar to 88% of our patient group. In a study on 32 TBM patients conducted by J Kalita et al., abnormal EEG was present in 75% of patients [11], which was in concordance with our study in which abnormal EEG was present in 55 patients (59.78%)

The Gupta et al. study reported the presence of altered mental status (AMS) and motor deficits as predictors of adverse outcomes in patients with TBM [12]. This was concordant with our study in which 21 patients (72.4%) with seizures presented in AMS, which was statistically significant (p 0.043), and 8 patients (27%) with seizures had decreased power in limbs, which was suggestive of focal neurological deficits (p 0.028).

On CNS imaging, the occurrence of exudates (p 0.034) and infarcts (p 0.031) in patients with seizures was statistically significant and infarcts were independent predictors of seizures (p 0.01), which is concordant with the UK Misra et al. study [6], in which the presence of lobar infarcts was significant in patients with seizures, and in contrast to the RS Kirar study that exhibited no association between cerebral images and poor outcomes [13]. In the Song et al. study, the most common imaging finding was meningeal enhancement followed by tuberculoma, and cortical involvement and epileptiform discharges were independent predictors of non-single seizures [5].

The pathophysiology of seizures in this study has been attributed to structural brain changes which include tuberculomas, cerebral infarcts, and hydrocephalus, which was similar to previous studies [6,13].

There was no significant difference in the mortality at the end of 3 months in patients with seizures (48.3%) and without seizures (40.8%), which was similar to the UK Misra et al. study [6], where mortality at the end of three and six months in patients of TBM with seizures did not significantly differ with or without seizures. In the Song et al. study, seizures were associated with poor functional outcomes at the end of 12 months [5].

In this study, the timing of seizures (early and late) and type of seizures (focal and GTCS) did not affect the functional outcome at the end of 3 months, whereas in the study by Song et al., these were associated with poor functional outcomes at the end of 12 months [5]. By the end of three months, seizure recurrence was seen only in two patients, as extensive evaluation and follow-up of the patients were done in this study.

Limitations

This study has shown no significant difference in outcomes between patients with and without seizures, as the follow-up was done for a short duration. Further studies are required with a larger number of patients and a long duration of follow-up to ascertain these results.

## Conclusions

In this study of TBM patients, seizures were highly prevalent. Patients with seizures had significantly decreased motor power on clinical examination, and basal exudates and cerebral vasculitic infarcts on CNS imaging were noted. Cerebral vasculitic infarcts were the independent predictor of seizures in our study. Seizures did not affect mortality at the end of three months in patients with TBM. Timing (early and late-onset) and type of seizures (GTCS and focal) did not affect the outcome or mortality at the end of three months. Early diagnosis and aggressive management, especially in patients presenting with high-risk features, are critical to improving survival and functional outcomes.
